# Propensity-Matched Analysis of Early and Long-Term Clinical Outcomes with Self-Expandable Prostheses in TAVR: Portico vs. CoreValve Evolut R

**DOI:** 10.3390/jcm14051523

**Published:** 2025-02-24

**Authors:** Uwe Primessnig, Julia M. Wiedenhofer, Sophie Berlinghof, Juliane Ducaruge, Tobias D. Trippel, Anna Brand, Sebastian Spethmann, Ulf Landmesser, Florian Blaschke, Simon H. Sündermann, Herko Grubitzsch, Volkmar Falk, Christoph Klein, Axel Unbehaun, Henryk Dreger, Mohammad Sherif

**Affiliations:** 1 Deutsches Herzzentrum der Charité, Department of Cardiology, Angiology and Intensive Care Medicine, Campus Virchow-Klinikum, 13353 Berlin, Germany; 2 Charité—Universitätsmedizin Berlin, Corporate Member of Freie Universität Berlin and Humboldt-Universität zu Berlin, 10117 Berlin, Germany; 3 DZHK (German Centre for Cardiovascular Research), 10785 Berlin, Germany; 4 Deutsches Herzzentrum der Charité, Department of Cardiology, Angiology and Intensive Care Medicine, Campus Charité Mitte, 10785 Berlin, Germany; 5 Deutsches Herzzentrum der Charité, Department of Cardiology, Angiology and Intensive Care Medicine, Campus Benjamin Franklin, 10785 Berlin, Germany; 6 Deutsches Herzzentrum der Charité, Department of Cardiothoracic and Vascular Surgery, 10785 Berlin, Germany

**Keywords:** transcatheter aortic valve replacement (TAVR), surgical valve replacement (SAVR), self-expandable (SE), balloon-expandable (BE)

## Abstract

**Background**: Transcatheter aortic valve replacement (TAVR) has emerged as a well-established option for patients with severe aortic stenosis who present high or extreme surgical risk. Direct comparisons of outcomes between different valve prostheses are important to assist operators in making an informed device selection. We aimed to perform a comparative analysis of early clinical outcomes at 30 days and long-term outcomes up to 3 years after TAVR using self-expandable Portico or CoreValve Evolut R valve prostheses. **Methods**: Out of 396 patients treated with either Portico or CoreValve Evolut R valves from January 2018 to December 2021, 79 patients were assigned to each group after 1:1 propensity score matching based on baseline parameters. Peri- and postprocedural outcomes at 30 days and up to a 3-year follow-up period were retrospectively collected according to the Valve Academic Research Consortium (VARC-2) criteria. **Results**: The immediate survival rate was 100% in both groups. The 30-day mortality was 0.0% in the Portico group and 1.3% in the CoreValve Evolut group (*p* = 1). Minor postprocedural bleeding was more frequent in the Evolut group both at 30 days (8.9% vs. 0%, *p* = 0.02) and at 3 years (11.4% vs. 3.8%, *p* = 0.133). There were no statistically significant differences regarding the combined safety endpoint (*p* = 1), acute kidney injury (AKIN 2 or AKIN 3) (*p* = 1; *p* = 0.477), or new pacemaker implantation (*p* = 0.31), at either 30 days or 3 years. Postprocedural myocardial infarction and stroke showed comparable rates in both groups. **Conclusions**: In terms of early clinical outcomes, no statistically significant differences were observed between the two groups of self-expandable valve prostheses, except for a significantly higher rate of minor bleeding in the Evolut group at 30 days. Notably, this trend of increased minor bleeding in the Evolut group persisted over the 3-year follow-up period, although the difference did not reach statistical significance. Both groups demonstrated low rates of all-cause mortality and clinical complications at long-term follow-up. The choice of valve should be customized to the individual characteristics of each patient.

## 1. Introduction

Transcatheter aortic valve replacement (TAVR) has become the preferred treatment approach for patients with symptomatic severe aortic stenosis who face a high perioperative risk of morbidity and mortality associated with surgical valve replacement (SAVR) [1,2]. Initially used for high-risk patients, TAVR has increasingly been extended to those at intermediate [3] and low surgical risk [4,5]. In intermediate-risk patients, the randomized PARTNER 2 trial demonstrated that TAVR offers clinical outcomes comparable to SAVR at 2 years [3] and at 5 years [6]. Similarly, the Evolut Low Risk trial, focusing on self-expandable (SE) valve prostheses in low-risk patients, showed favorable outcomes at the 3-year follow-up [7] after previously demonstrating non-inferiority at 2 years [8]. The 5-year follow-up of the PARTNER 3 trial also confirmed a similar incidence of clinical endpoints in low-risk patients [5], with significantly favorable outcomes in mortality, stroke, and rehospitalization at 1-year [9] and 2-year [10] follow-ups. These findings further expanded the role of TAVR in broader patient populations.

Given these outcomes, attention has also focused on comparing the performance of different transcatheter valve types. SE and balloon-expandable (BE) valves represent the two primary types of transcatheter heart valves used for TAVR. The multicenter randomized CHOICE trial demonstrated increased device success and lower rates of pacemaker implantation with BE valves at 30-day evaluation [11]. However, at 1 year [11] and 5 years [12], no significant differences were observed between the two valve types. At 5 years, SE valves demonstrated superior forward-flow hemodynamics [12], suggesting potential long-term advantages for certain patients. Beyond comparing SE and BE valves, evaluating different SE valve devices is essential for optimizing patient outcomes. The CoreValve Evolut R (Medtronic, Minneapolis, MN, USA) is a supra-annular SE valve prosthesis featuring a multilevel, radiopaque nitinol stent frame with a diamond cell configuration. The stent frame has different sections with varying radial force properties, allowing anchoring, resistance to changes in shape/diameter, and orientation within blood flow [13]. In contrast, the Portico valve (Abbott Structural Heart, St Paul, MN, USA) is a SE prosthesis featuring large, open cells in a nitinol stent frame with bovine pericardial leaflets positioned intra-annularly. The re-sheathable design enables the valve to be repositioned during the procedure [14]. An overview of both valve prostheses is provided in Table 1.

The present study directly compares the SE valve devices Portico and CoreValve Evolut R in terms of clinical outcomes and efficacy at a 30-day and 3-year follow-up.

## 2. Materials and Methods

A retrospective analysis was conducted on data from 1901 patients who underwent TAVR at Charité University Medical Center between January 2018 and December 2021. Baseline characteristics, endpoints, and matching variables underwent complete case analysis, and cases with missing data for these variables were excluded from further analysis. No imputation was performed for variables with missing data below the 5% threshold. Patients receiving valve prostheses other than the Portico or CoreValve Evolut R or underwent TAVR via a non-transfemoral access site were excluded from the analysis. To achieve an unbiased comparison, propensity score matching was used to match patients who received Portico valves with an equal number of CoreValve Evolut R recipients. The matching quality was confirmed by calculating the standardized mean differences (SMD), indicating a well-matched cohort.

The indication for TAVR was assessed by the institutional heart team. The selection of the valve type was based on the expertise of the operator and the heart team. The baseline surgical risk was estimated using EuroScore II and STS-score. All patients underwent transesophageal echocardiography and transthoracic echocardiography within six weeks prior to the procedure for preprocedural screening. In cases where additional assessment was needed, computed tomography was performed to assist with access site planning. This was the institutional standard of practice at the time the transcatheter aortic valve implantation procedures were performed. During a 30-day, 1-year, 2-year, and 3-year follow-up period, the clinical outcomes were evaluated in accordance with the Valve Academic Research Consortium-2 (VARC-2) consensus document [15].

The primary endpoint was early and long-term safety outcomes after a 3-year follow-up, based on the VARC-2 composite, which included all-cause mortality, stroke (both disabling and non-disabling), life-threatening bleeding, acute kidney injury (AKIN stage 2 or 3), coronary obstruction requiring intervention, major vascular complications, and valve-related dysfunction requiring repeat procedures. Secondary endpoints included individual clinical outcomes such as all-cause mortality, stroke (disabling and non-disabling), bleeding (minor, major, and life-threatening), acute kidney injury (AKIN stage 2 or 3), coronary obstruction requiring intervention, major vascular complications, valve-related dysfunction requiring repeat procedures, new atrioventricular block, and new pacemaker implantation.

The study adhered to the STROBE guidelines (Strengthening the Reporting of Observational Studies in Epidemiology) to ensure transparent reporting. All analyses were conducted on de-identified datasets to maintain patient confidentiality. Ethical approval was obtained from the Charité’s ethics committee (EA4/131/23).

### Statistical Analysis

Propensity score analysis was performed using a logistic regression model that included baseline patient characteristics such as age, sex, body mass index (BMI), arterial hypertension, diabetes, atrial fibrillation, renal insufficiency, and NYHA class III and IV. A 1:1 nearest neighbor matching was then performed between the Portico and CoreValve Evolut R groups. Matching was performed with a caliper width of 0.1 standard deviations of the logit of the propensity score. This matching process yielded standardized mean differences below 0.10 for each covariate, indicating a well-balanced matching. For the analysis of differences between the two valve groups, Student’s *t*-test or the Wilcoxon–Mann–Whitney U-test were applied to continuous variables based on normality, with normality assessed using the Shapiro–Wilk test. The categorical variables were compared using the Chi-square test. The continuous variables are reported as means (± standard deviations) or medians [interquartile ranges], while categorical variables are presented as counts and percentages. All statistical analyses were conducted using R 4.2.3. (R Core Team 2023).

## 3. Results

**Figure 1:**
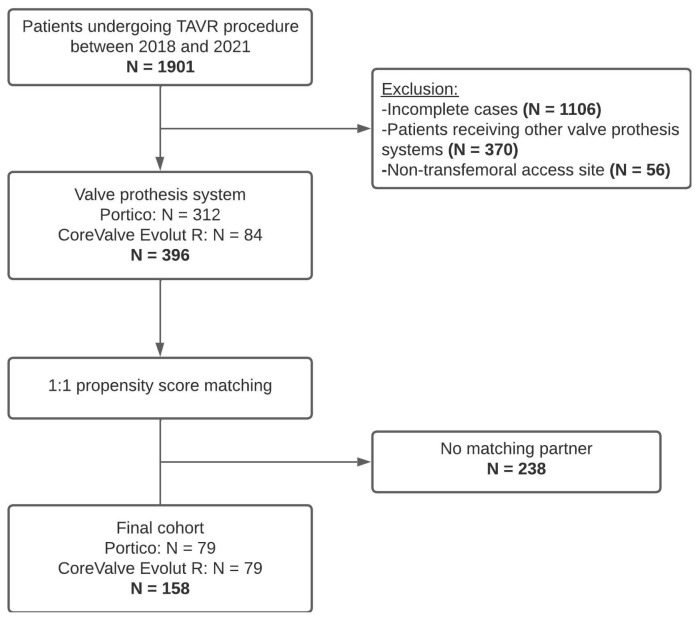
Study selection process.

After complete case analysis, 1106 out of the initial 1901 patients who underwent TAVR were excluded. Additionally, 46.5% (n = 370) of the 795 remaining patients did not receive the valve prosthesis included in the study and 7.0% (n = 56) underwent TAVR via a non-transfemoral access site. This left a remaining sample size of 396 patients, out of which 158 patients were successfully matched. Among the matched patients, 79 patients received a Portico valve, and 79 patients received a CoreValve Evolut R. Figure 1 presents the study population selection process.

### 3.1. Baseline Characteristics

Baseline characteristics of the matched patient population are displayed in Table 2 and were well-balanced between Portico and Evolut groups. There were no significant differences in the demographic characteristics, comorbidities, or echocardiographic parameters between the two groups. The baseline AVA was 0.75 cm^2^ (0.15) in the Portico group and 0.81 cm^2^ (0.25) in the Evolut group (*p* = 0.28). The indexed AVA was 0.40 cm^2^/m^2^ (0.09) and 0.44 cm^2^/m^2^ (0.14), respectively (*p* = 0.081). The aortic valve mean gradients were 41.1 (15.3) mmHg and 41.2 (14.9) mmHg (*p* = 0.826). The left ventricular end-diastolic internal diameter (LVEDD) fell within the normal range for both the Portico and Evolut groups with values of 44.0 (8.32) mm and 44.3 (6.09) mm (*p* = 0.605). Both the Portico and Evolut group showed left atrial dilation with mean diameters of 43.7 (6.65) mm and 46.7 (8.17) mm (*p* = 0.248). Patients who received a CoreValve Evolut R valve also demonstrated a mildly increased mean systolic pulmonary artery pressure with 40.0 (15.1) mmHg compared to 38.2 (15.4) mmHg in the Portico group (*p* = 0.402). The median EuroScore II was calculated as 4.23% [1.15–40.7] in the Portico group and 4.3% [1.10–47.5] in the Evolut group (*p* = 0.809). The median STS-score was increased in the Evolut group with 4.38% [1.34, 24.1] compared to 3.84% [1.04, 16.5] in the Portico group (*p* = 0.225).

### 3.2. Procedure Details

Procedural details are displayed in Table 3. In both valve groups, the immediate post-intervention survival rate was 100%. In the Evolut group, 7.6% (n = 6) of procedures were performed as valve-in-valve TAVR, while no valve-in-valve TAVR procedures were performed in the Portico group (*p* = 0.037). The procedural time was significantly prolonged in the Evolut group compared to the Portico group. The mean procedural time was 94.3 (38.8) min for the Portico group and 78.7 (31.3) min for the Evolut group (*p* = 0.006). However, the Portico group showed a higher frequency of relevant transvalvular regurgitation compared to the Evolut group (7.6% (n = 6) vs. 2.5% (n = 2); *p* = 0.276). Mild and moderate paravalvular leakage (PVL) did not differ significantly between both groups. Other parameters such as contrast medium use, total radiation time or dose, balloon valvuloplasty, or total hospital length of stay did not show any significant differences between the two groups. In the Portico group, the 29 mm and 27 mm valve sizes were the most frequently used, each accounting for 34.2% (n = 27) of cases. In the Evolut group, the 29 mm valve size was the most common, used in 49.4% (n = 39) of cases. A detailed valve size distribution is provided in Supplementary Table S1.

### 3.3. Primary and Secondary Safety Outcomes at 30 Days

No statistically significant differences were found in the primary safety composite endpoint at 30-days early clinical follow-up. Concerning secondary single endpoints, the incidence of minor postprocedural bleeding was significantly higher in the Evolut group compared to the Portico group, with rates of 8.9% versus 0% (*p* = 0.02). There were no significant differences in major bleeding between both groups (*p* = 1) and no occurrence of life-threatening bleeding in both groups. There were no 30-day mortality events in the Portico group, while the Evolut group had a 1.3% (n = 1) 30-day mortality rate (*p* = 1). There was one case of access-related complications in the Portico group, which involved delayed wound healing at the puncture site, requiring long-term follow-up. No major vascular complications, coronary artery obstruction, or valve-related dysfunction were reported in either the Portico or the Evolut groups.

### 3.4. Primary and Secondary Long-Term Safety Outcomes

Concerning the primary safety endpoint, the rate of VARC-2 safety composite was slightly higher in the CoreValve Evolut group (6.3%) compared to the Portico group (5.1%), but this difference was not statistically significant (*p* = 1). Similarly, no significant differences were observed among the secondary safety endpoints. The 3-year all-cause mortality rate was 3.8% in the Portico group compared to 1.3% in the Evolut group (*p* = 0.613). The proportion of patients requiring new pacemaker implantation was slightly higher in the Portico group than in the Evolut group (24.1% vs. 17.7%; *p* = 0.434). The rate of new atrioventricular blocks was identical between the groups at 27.8% (*p* = 1). There were three cases of non-disabling or disabling stroke reported in the Evolut group over three years, whereas no strokes were reported in the Portico group. Minor bleeding rates were higher in the Evolut group at long-term follow-up (11.4%) compared to the Portico group (3.8%), although this difference was not statistically significant (*p* = 0.133). Furthermore, one case of major bleeding and one case of life-threatening bleeding occurred in the Evolut group, while no additional cases of major or life-threatening bleeding were observed in the Portico group over the 3-year follow-up period. There was a trend toward a higher overall rehospitalization rate in the CoreValve Evolut group (31.6%) compared to the Portico group (17.7%) (*p* = 0.065). Among rehospitalized patients, cardiac decompensation accounted for 6.3% (n = 5) of cases in the Evolut group and 5.1% (n = 4) in the Portico group (*p* = 1). Additionally, one non-ST elevation myocardial infarction requiring coronary stent implantation occurred in the Evolut group during the second year after TAVR. In the Portico group, one patient experienced an out-of-hospital cardiac arrest of unclear etiology during the third year of follow-up and was successfully resuscitated. Within the first year after TAVR, three additional cases of access-related complications were reported: two in the Evolut group and one in the Portico group. All were related to access-site associated aneurysms, in one case occurring with minor bleeding. No cases of major vascular complications were reported. After TAVR, three patients underwent additional valve interventions, including two MitraClip procedures and one TriClip procedure. No interventions on the aortic valve were reported during the long-term follow-up. Within the 3-year follow-up period, 5.1% (n = 4) of patients in the Portico group and 3.8% (n = 3) in the Evolut group required coronary angiography. A detailed summary of early and long-term clinical outcomes is provided in Table 4.

### 3.5. Postprocedural Echocardiography

After TAVR, LVEF remained within normal range for both groups but significantly improved in the Portico group, with a mean increase of Δ + 2.3 (7.7) % (*p* = 0.028), and in the Evolut group, with a mean increase of Δ + 2.2 (8.8) % (*p* = 0.049) compared to pre-TAVR values. Mean aortic valve gradients and peak aortic jet velocity (AV Vmax) were effectively reduced in both groups. In the Portico group, the values decreased from 41.1 (15.3) mmHg to 8.53 (3.7) mmHg and from 3.91 (0.87) m/s to 2.00 (0.48) m/s (both *p* < 0.0001). Similarly, in the Evolut group, the mean gradients decreased from 41.2 (14.9) mmHg to 10.8 (7.6) mmHg and the AV Vmax decreased from 3.94 (0.76) m/s to 2.18 (0.68) m/s (both *p* < 0.0001). Although the decrease in left atrial diameter in the Portico group (Δ − 3.7 (5.9) mm) to 41.7 (5.3) mm was not statistically significant, the post-TAVR left atrial diameter was significantly smaller in the Portico group compared to the Evolut group, which remained at 46.6 (7.47) mm (*p* = 0.048). For all other echocardiographic parameters, no significant differences were observed between the two valve groups.

**Figure 2:**
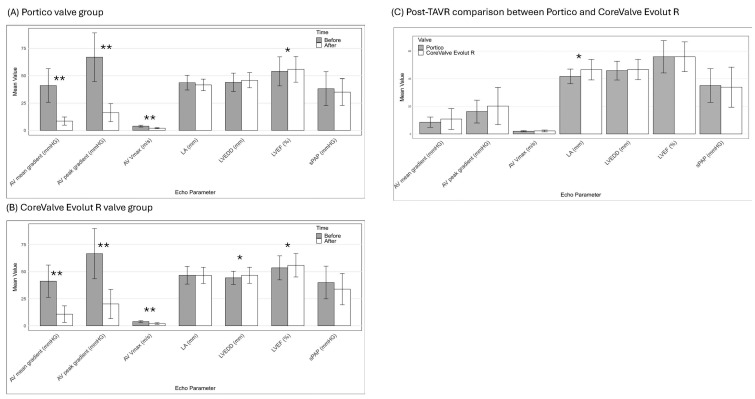
Comparison of echocardiographic parameters before and after TAVR procedure within (**A**) Portico valve group and (**B**) CoreValve Evolut R valve group. A direct comparison of echocardiographic parameters between Portico and CoreValve Evolut R groups after TAVR is provided in (**C**). AV, aortic valve; AV Vmax, peak aortic jet velocity; LA, left atrium diameter; LVEF, left ventricular ejection fraction; LVEDD, left ventricular end-diastolic internal diameter; sPAP, pulmonary artery systolic pressure; TAVR, transcatheter aortic valve replacement. * Significant, *p* < 0.05; ** Highly significant, *p* ≤ 0.01.

Figure 2 shows echocardiographic comparisons before and after the TAVR procedure. Panel A displays changes in the Portico valve group, while Panel B shows changes in the CoreValve Evolut R group. Panel C provides a direct comparison of post-TAVR echocardiographic parameters between the two valve groups. Detailed information on pre- and post-echocardiographic measurements within both valve groups is provided in Supplementary Table S2.

## 4. Discussion

In this comparison of early-generation and new-generation SE valve prostheses, we observed a significant difference in minor postprocedural bleeding rates, with the Evolut group showing a higher incidence than the Portico group at 30 days. However, no significant differences were noted in the combined VARC-2 safety endpoint at 30-days or after the 3-year follow-up period. Additionally, the Evolut group had significantly longer procedural times. To our knowledge, this is the first direct case-matched comparison of the Portico and CoreValve Evolut R SE valves.

In the 2020 PORTICO IDE trial, the Portico valve was compared to other commercially available BE and SE valves, including the CoreValve Evolut R. The Portico valve was associated with higher 30-day composite safety endpoint rates [14]. In contrast, our study found similar composite safety endpoint rates between the Portico and Evolut groups (3.8% vs. 3.8%). There was no significant difference in all-cause mortality at 30 days, with no death in the Portico group, while the Evolut group had one. At 3 years, the mortality rate in the Portico group increased to 3.8%, while the Evolut group had no additional cases, maintaining a rate of 1.3%. Mortality rates were in line with previously reported rates for both valve groups [14,16,17,18].

The most frequent secondary complication observed at both 30-day and 3-year follow-ups was the development of a new atrioventricular block, with comparable rates between groups. New pacemaker implantation was the second most common complication, with slightly higher rates in the Portico group compared to the Evolut group without being statistically significant. Generally, SE valve prostheses are associated with higher rates of new pacemaker implantations compared to BE valves. However, recent years have shown a trend toward reduced pacemaker implantation needs for both BE and SE devices. In 2018, van Rosendael et al. reported a range of 16.3% to 37.7% for the occurrence of new pacemaker implantation with the early-generation CoreValve Evolut [19]. Thiele et al., 2020, reported a rate of 23% new permanent pacemaker implantation in patients who received the second-generation Evolut R device as part of the randomized controlled SOLVE-TAVI trial with a 30-day follow-up [20]. Compared to these reported rates of new pacemaker implantation in SE valves, the observed rate of 15.2% at 30 days and 17.7% at 3 years for the CoreValve Evolut device in our study is relatively low. However, considering the notably broad range reported by van Rosendael et al., 2018, alongside an implantation rate of 18% for patients receiving the Evolut R device in He et al., 2019, our findings are well situated within this variability [19,21].

According to a recent systematic review by Jaiswal et al., 2023, which assessed the procedural safety of the Portico valve, the incidence of early new pacemaker implantations was reported at 15.7% [22]. Our study’s rate of new pacemaker implantations for the Portico valve group, at 22.8%, is slightly increased compared with this figure. Despite the great interest in the specific indication for new pacemaker implantations, there were no available data for this aspect within our study.

In terms of postprocedural bleeding, the Evolut group demonstrated a significantly higher rate of minor bleeding events compared to the Portico group in the early postprocedural period. Although minor bleeding remained more frequent in the Evolut group during long-term follow-up, the difference was not statistically significant. The observational, multicenter FORWARD study by Grube et al., 2016, reported 30-day minor and major bleeding rates of 2.4% and 6.2%, respectively, for the CoreValve Evolut R [23]. In comparison, our study found higher rates of minor bleeding at 8.9% and lower rates of major bleeding at 1.3%. Only in the Evolut group one case of life-threatening bleeding was reported within 1 year after TAVR. Notably, the very low rate of life-threatening bleedings in the Portico group contrasts with the 5.9% rate reported by Makkar et al., 2020 [14]. Additionally, the early and long-term major bleeding rates of 2.5% in the Portico group were slightly lower than the 3.8% reported by Mas-Peiro et al. in 2019 [14,18].

No significant differences in transvalvular regurgitation were observed between the two valve groups, consistent with the findings of the PORTICO IDE trial, which also reported no significant differences in moderate or greater PVL at 30-day follow-up. However, we found a relatively high rate of moderate PVL at the 30-day follow-up within the Portico group compared to the PORTICO IDE trial and Mas-Peiro et al., 2019 (13.9% vs. 6.1% and 8.2%) [14]. The occurrence of moderate PVL in the Evolut group was comparable with previously reported rates [11,24].

Postprocedural echocardiographic findings demonstrate excellent outcomes for both valve groups, particularly in terms of mean aortic valve gradients and AV Vmax. Both valve groups showed notable improvements in these parameters, indicating an effective treatment of aortic valve stenosis and improved hemodynamics. Despite longer procedural times in the Evolut group, there were no differences in contrast medium use or radiation time and dose. The mean contrast medium use was lower in our study compared to previous studies, with 120 mL compared to 160 mL for the Portico valve [18] and 119 mL compared to 132 mL for the CoreValve Evolut R [17].

The CoreValve US Pivotal clinical trial was the first study comparing TAVR with SE valves to SAVR in high-risk patients [24], while the subsequent SURTAVI trial proved the clinical efficacy of SE valves in patients with intermediate risk [25]. Since then, several head-to-head comparisons between the CoreValve Evolut and BE valves have been conducted, but only a limited number of studies have directly compared the CoreValve Evolut to other self-expandable valve prostheses, except for comparison with its own iterations, Evolut R and Evolut Pro [11,23,26,27]. However, there is a lack of real-world data focusing on direct SE valve comparisons. Ivanov et al. recently conducted a case-matched comparison of the CoreValve Evolut and ACURATE neo valve prostheses, showing good performance and comparable clinical outcomes for both valves [16]. Until now, no study has directly compared the Portico valve and the CoreValve Evolut using real-world data from a high-volume center.

## 5. Limitations

While this study’s strengths are the reflection of current clinical practice in real-world data in a high-volume center in Germany together with the reduction of potential confounders and the achievement of comparable groups by propensity score matching, there are still limitations to consider. Results should be carefully interpreted, as there might be potential biases and unidentified confounding factors introduced by the retrospective design. Moreover, as this study represents the clinical practice of a single high-volume center only, the findings need to be confirmed in a multicenter study.

Although the exclusion of a substantial portion of patients due to incomplete records has potentially limited the generalizability of our findings, it was necessary to ensure a well-balanced and robust matching process, thereby enhancing the validity of our comparative analysis.

The analyses were conducted between the Portico and CoreValve Evolut valves, for which newer generations are now available. Despite the introduction of these newer iterations, which largely retain the core functionalities and design principles of their predecessors, there remains a significant gap in real-world data directly comparing the performance of these and other current self-expandable devices. The availability of such comparative data is very valuable for future studies and informed clinical decision-making. This study involved patients who were enrolled from 2018 to 2021, and it was based on the VARC-2 criteria, which were the standard guidelines during that period. Despite the publication of the VARC-3 criteria in 2021, applying them retrospectively was not possible due to limitations in the available data.

## 6. Conclusions

In this case-matched analysis between the Portico and CoreValve Evolut R SE valve prostheses, apart from an increased risk of minor postprocedural bleeding in the Evolut group at 30 days, no statistically significant differences were found in early and long-term clinical safety outcomes. However, the longer procedural times observed when using the CoreValve Evolut R should be considered in patients with higher periprocedural risk.

## Figures and Tables

**Table 1:** Comparsion of characteristics of Portico™ (Abbott Structural Heart, St Paul, MN, USA) and CoreValve^®^ Evolut™ R Valve (Medtronic, Minneapolis, MN, USA).

Characteristic	Portico™ (Abbott Structural Heart, St Paul, MN, USA)	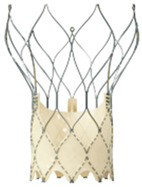	CoreValve® Evolut™ R Valve (Medtronic, Minneapolis, MN, USA)	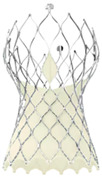
Valve material	Nitinol stent, bovine pericardial leaflets, porcine pericardial sealing cuff		Nitinol stent, tricuspid porcine pericardial leaflets	
Valve frame	Self-expanding nitinol		Self-expanding nitinol	
Leaflet position	Intra-annular		Supra-annular	
Delivery system and valve sizes	FlexNav™ Delivery System: transfemoral, transaortic or transsubclavian access; 18 F (23 mm, 25 mm), 19 F (27 mm, 29 mm)		EnVeo™ R Delivery System: transfemoral, transaortic or transsubclavian access; 14 F outer diameter (23 mm, 26 mm, 29 mm, 34 mm)	
Minimum vessel diameter	≥6.0 mm for 23 mm and 25 mm valves, ≥6.5 mm for 27 mm and 29 mm valves		≥5.0 mm for 23–29 mm valves, ≥5.5 mm for 34 mm valve	
Repositionability	Re-sheathable and repositioning up to 2 times before full deployment		Recapturable and repositionable before 80% deployment	
Anticalcification treatment	Linx™ anticalcification technology for leaflets and cuff		None specified	

**Table 2:** Baseline characteristics before and after matching process.

		Unmatched			Matched		
		Portico (N = 312)	CoreValve Evolut (N = 84)	*p*-Value	Portico (N = 79)	CoreValve Evolut (N = 79)	*p*-Value
Female		194 (62.2%)	40 (47.6%)	0.022 *	37 (46.8%)	39 (49.4%)	0.873
Age		82.5 (5.20)	80.7 (5.46)	0.002 **	80.7 (5.23)	81.0 (5.04)	0.834
BMI (kg/m^2^)		26.7 (5.12)	26.2 (5.14)	0.477	27.2 (4.96)	26.3 (5.14)	0.289
Obesity (BMI ≥ 30)		82 (26.3%)	20 (23.8%)	0.749	21 (26.6%)	19 (24.1%)	0.855
NYHA I & II		67 (21.5%)	19 (22.6%)	0.939	16 (20.3%)	18 (22.8%)	0.846
NYHA III & IV		245 (78.5%)	65 (77.4%)	0.939	63 (79.7%)	61 (77.2%)	0.846
Arterial hypertension		277 (88.8%)	70 (83.3%)	0.246	67 (84.8%)	67 (84.8%)	1
Diabetes mellitus		95 (30.4%)	21 (25.0%)	0.402	25 (31.6%)	20 (25.3%)	0.481
Previous coronary heart disease		195 (62.5%)	54 (64.3%)	0.862	48 (60.8%)	51 (64.6%)	0.742
History of atrial fibrillation		129 (41.3%)	32 (38.1%)	0.679	29 (36.7%)	30 (38.0%)	1
History of renal insufficiency		123 (39.4%)	33 (39.3%)	1	33 (41.8%)	31 (39.2%)	0.871
EuroScore II (%)		4.30 [0.670, 44.7]	4.35 [1.10, 47.5]	0.7	4.23 [1.15, 40.7]	4.30 [1.10, 47.5]	0.809
STS-Score (%)		4.25 [0.998, 29.5]	4.20 [1.34, 26.5]	0.854	3.84 [1.04, 16.5]	4.38 [1.34, 24.1]	0.225
Preprocedural assessment							
Preprocedural rhythm	Atrial fibrillation	72 (24.5%)	8 (10.3%)	0.01 *	14 (18.2%)	8 (10.8%)	0.292
	Atrioventricular block	37 (12.6%)	14 (17.9%)	0.304	6 (7.8%)	14 (18.9%)	0.076
	Bundle branch block	79 (27.1%)	21 (27.3%)	1	24 (31.6%)	21 (28.8%)	0.845
LVEF (%)		54.9 (10.7)	53.7 (11.5)	0.604	54.0 (13.2)	53.5 (11.1)	0.471
Aortic valve area (cm^2^)		0.75 (0.16)	0.82 (0.25)	0.085	0.75 (0.15)	0.81 (0.25)	0.28
Aortic valve area index (cm^2^/m^2^)		0.41 (0.01)	0.45 (0.14)	0.061	0.40 (0.09)	0.44 (0.14)	0.081
Aortic valve mean gradient (mmHG)		40.8 (14.5)	40.8 (15.2)	0.854	41.1 (15.3)	41.2 (14.9)	0.826
Aortic valve peak gradient (mmHG)		64.5 (22.3)	65.5 (23.9)	0.821	67.0 (22.2)	66.6 (23.2)	0.856
AV Vmax (m/s)		4.23 (3.59)	3.89 (0.822)	0.588	3.91 (0.866)	3.94 (0.764)	0.895
LVEDD (mm)		43.5 (7.10)	44.5 (6.73)	0.602	44.0 (8.32)	44.3 (6.09)	0.605
sPAP (mmHG)		37.2 (12.9)	39.9 (15.3)	0.379	38.2 (15.4)	40.0 (15.1)	0.402
LA (mm)		43.1 (7.19)	46.2 (7.82)	0.151	43.7 (6.65)	46.7 (8.17)	0.248

Values are displayed as frequencies (percent), mean (standard deviation) and median [interquartile range]. AV Vmax = peak aortic jet velocity; BMI = Body mass index; LA = left atrium diameter; LVEF = left ventricular ejection fraction; LVEDD = left ventricular end-diastolic internal diameter; sPAP = pulmonary artery systolic pressure; NYHA = New York Heart Association Classification of Heart Failure. * Significant, *p* < 0.5; ** Highly significant, *p* ≤ 0.01.

**Table 3:** Procedural details.

		Portico (N = 79)	CoreValve Evolut (N = 79)	*p*-Value
Total procedural time (min)		78.7 (31.3)	94.3 (38.8)	0.006 **
Contrast medium use (ml)		120 (56.5)	119 (59.9)	0.846
Total radiation time (min)		13.1 (7.09)	14.7 (7.73)	0.208
Radiation dose (Gy/cm^2^)		43.7 (35.7)	42.9 (32.9)	0.934
Anesthetic technique	Conscious sedation anesthesia	20 (26.7%)	13 (18.3%)	0.313
	Laryngeal mask airway	1 (1.3%)	0 (0%)	1
	Endotracheal intubation	54 (72.0%)	58 (81.7%)	0.235
Valve size (mm)		26.8 (2.04)	27.6 (2.72)	0.079
Paravalvular leakage	Mild	36 (45.6%)	29 (36.7%)	0.332
	Moderate	11 (13.9%)	6 (7.6%)	0.304
Final aortic regurgitation		6 (7.6%)	2 (2.5%)	0.276
Post-TAVR balloon valvuloplasty		47 (59.5%)	47 (59.5%)	1
Valve-in-valve TAVR		0 (0%)	6 (7.6%)	0.037 *
Total hospital length of stay (days)		8.00 [4.00, 47.0]	8.00 [4.00, 45.0]	0.801

Values are displayed as frequencies (percent), mean (standard deviation) and median [interquartile range]. TAVR = transcatheter aortic valve replacement. * Significant, *p* < 0.5; ** Highly significant, *p* ≤ 0.01.

**Table 4:** Early clinical outcomes (30-day) and long-term clinical outcomes (3 years).

		Portico (N = 79)	CoreValve Evolut (N = 79)	*p*-Value
30-day follow-up				
All-cause mortality		0 (0%)	1 (1.3%)	1
Postprocedural myocardial infarct	<72 h	0 (0%)	1 (1.3%)	1
	>72 h	0 (0%)	0 (0%)	na
Stroke (non-disabling or disabling)		0 (0%)	2 (2.5%)	0.477
Postprocedural bleeding	Minor	0 (0%)	7 (8.9%)	0.02 *
	Major	2 (2.5%)	1 (1.3%)	1
	Life-threatening	0 (0%)	0 (0%)	na
Major vascular complication		0 (0%)	0 (0%)	na
Access site complication		1 (1.3%)	0 (0%)	1
Acute kidney injury	Stage 1	3 (3.8%)	1 (1.3%)	0.613
	Stage 2	1 (1.3%)	0 (0%)	1
	Stage 3	2 (2.5%)	0 (0%)	0.477
New atrioventricular block		19 (24.1%)	20 (25.3%)	1
New pacemaker implantation		18 (22.8%)	12 (15.2%)	0.31
VARC-2 early safety composite		3 (3.8%)	3 (3.8%)	1
3-year follow-up				
All-cause mortality		3 (3.8%)	1 (1.3%)	0.613
Rehospitalization		14 (17.7%)	25 (31.6%)	0.065
Myocardial infarct		0 (0%)	2 (1.3%)	0.477
Cardiac decompensation		4 (5.1%)	5 (6.3%)	1
Resusication		1 (1.3%)	0 (0%)	1
Transient ischemic attack		0 (0%)	2 (2.5%)	0.477
Stroke (non-disabling or disabling)		0 (0%)	3 (3.8%)	0.244
Postprocedural bleeding	Minor	3 (3.8%)	9 (11.4%)	0.133
	Major	2 (2.5%)	2 (2.5%)	1
	Life-threatening	0 (0%)	1 (1.3%)	1
Major vascular complication		0 (0%)	0 (0%)	na
Access site complication		2 (2.5%)	2 (2.5%)	1
Acute kidney injury	Stage 1	5 (6.3%)	6 (7.6%)	1
	Stage 2	0 (0%)	2 (2.5%)	0.477
	Stage 3	1 (1.3%)	1 (1.3%)	1
New atrioventricular fibrillation		5 (6.3%)	5 (6.3%)	1
New atrioventricular block		22 (27.8%)	22 (27.8%)	1
New pacemaker implantation		19 (24.1%)	14 (17.7%)	0.434
Valve intervention		1 (1.3%)	2 (2.5%)	1
VARC-2 early safety composite		4 (5.1%)	5 (6.3%)	1

Values are displayed as frequencies (percent), mean (standard deviation) and median [interquartile range]. TAVR = transcatheter aortic valve replacement. * Significant, *p* < 0.5.

## Data Availability

The authors will provide the raw data of this article without further reservation.

## Supplementary Materials

The following supporting information can be downloaded at: https://www.mdpi.com/article/10.3390/jcm14051523/s1, Table S1. Implanted valve sizes. Table S2. Comparsion of echocardiographic parameters before and after TAVR procedure within Portico valve group and CoreValve Evolut R valve group.
