# Supplementary material for: Propensity-Matched Analysis of Early and Long-Term Clinical Outcomes with Self-Expandable Prostheses in TAVR: Portico vs. CoreValve Evolut R
Source: J Clin Med. 2025 Feb 24;14(5):1523. doi: 10.3390/jcm14051523 (PMC11900177; doi:10.3390/jcm14051523)
Supplement: Supplementary file 1 [file jcm-14-01523-s001.zip › jcm-3434058-supplementary.pdf]

Supplementary Table S1. Implanted valve sizes.

Valve size (mm)	Portico (N=79)	CoreValve Evolut (N=79)
23	10 (12.7%)	11 (13.9%)
25	15 (19.0%)	/
26	/	24 (30.4%)
27	27 (34.2%)	/
29	27 (34.2%)	39 (49.4%)
34	/	5 (6.3%)

Values are displayed as frequencies (percent).

Supplementary Table S2. Comparsion of echocardiographic parameters before and after TAVR procedure within Portico valve group and CoreValve Evolut R valve group.

**Portico (N=79)**	**Before TAVR**	**After TAVR**	**P-Value**
LVEF (%)	54.0 (13.2)	55.9 (11.7)	0.028*
Aortic valve mean gradient (mmHG)	41.1 (15.3)	8.53 (3.72)	<0.0001**
Aortic valve peak gradient (mmHG)	67.0 (22.2)	16.2 (8.28)	<0.0001**
AV Vmax (m/s)	3.91 (0.87)	2.00 (0.48)	<0.0001**
LVEDD (mm)	44.0 (8.32)	45.9 (6.84)	0.327
sPAP (mmHG)	38.2 (15.4)	35.1 (12.2)	0.052
LA (mm)	43.7 (6.65)	41.7 (5.32)	0.061
**CoreValve Evolut R (N=79)**			
LVEF (%)	53.5 (11.1)	55.9 (10.8)	0.049*
Aortic valve mean gradient (mmHG)	41.2 (14.9)	10.8 (7.61)	<0.0001**
Aortic valve peak gradient (mmHG)	66.6 (23.2)	20.2 (13.5)	<0.0001**
AV Vmax (m/s)	3.94 (0.764)	2.18 (0.675)	<0.0001**
LVEDD (mm)	44.3 (6.09)	46.7 (7.41)	0.01*
sPAP (mmHG)	40.0 (15.1)	33.9 (14.5)	0.188
LA (mm)	46.7 (8.17)	46.6 (7.47)	0.815

Values are displayed as frequencies (percent), mean (standard deviation) and median [interquartile range]. AV Vmax, peak aortic jet velocity; LA, left atrium diameter; LVEF, left ventricular ejection fraction; LVEDD, left ventricular end-diastolic internal diameter; sPAP, pulmonary artery systolic pressure. * Significant, p<0.5; ** Highly significant, p≤0.01.
